# Long Non-coding RNAs Associated With Neurodegeneration-Linked Genes Are Reduced in Parkinson’s Disease Patients

**DOI:** 10.3389/fncel.2019.00058

**Published:** 2019-02-22

**Authors:** Maximilianos Elkouris, Georgia Kouroupi, Alexios Vourvoukelis, Nikolaos Papagiannakis, Valeria Kaltezioti, Rebecca Matsas, Leonidas Stefanis, Maria Xilouri, Panagiotis K. Politis

**Affiliations:** ^1^Center for Basic Research, Biomedical Research Foundation of the Academy of Athens, Athens, Greece; ^2^Laboratory of Cellular and Molecular Neurobiology, Hellenic Pasteur Institute Athens, Greece; ^3^Center of Clinical Research, Experimental Surgery and Translational Research, Biomedical Research Foundation of the Academy of Athens, Athens, Greece; ^4^First Department of Neurology, National and Kapodistrian University of Athens Medical School, Athens, Greece

**Keywords:** long-non coding RNAs, Parkinson’s disease, alpha-synuclein, LRRK2, *Substantia Nigra*, exosomes

## Abstract

Transcriptome analysis has identified a plethora of long non-coding RNAs (lncRNAs) expressed in the human brain and associated with neurological diseases. However, whether lncRNAs expression levels correlate with Parkinson’s disease (PD) pathogenesis remains unknown. Herein, we show that a number of lncRNA genes encompassing transcriptional units in close proximity to PD-linked protein-coding genes, including *SNCA*, *LRRK2*, *PINK1*, *DJ-1*, *UCH-L1*, *MAPT* and *GBA1*, are expressed in human dopaminergic cells and post-mortem material, such as cortex, *Substantia Nigra* and cerebellum. Interestingly, these lncRNAs are upregulated during neuronal differentiation of SH-SY5Y cells and of dopaminergic neurons generated from human fibroblast-derived induced pluripotent stem cells. Importantly, six lncRNAs are found under-expressed in the nigra and three in the cerebellum of PD patients compared to controls. Simultaneously, *SNCA* mRNA levels are increased in the nigra, while *LRRK2* and *PINK1* mRNA levels are decreased both in the nigra and the cerebellum of PD subjects compared to controls, indicating a possible correlation between the expression profile of the respective lncRNAs with their adjacent coding genes. Interestingly, all dysregulated lncRNAs are also detected in human peripheral blood mononuclear cells and four of them in exosomes derived from human cerebrospinal fluid, providing initial evidence for their potential use as diagnostic tools for PD. Our data raise the intriguing possibility that these lncRNAs may be involved in disease pathogenesis by regulating their neighboring PD-associated genes and may thus represent novel targets for the diagnosis and/or treatment of PD or related diseases.

## Introduction

Exploration of non-coding genome has uncovered a growing list of formerly unknown regulatory lncRNAs with critical roles in the pathophysiology of many neurological diseases, including schizophrenia, bipolar disorder, cerebral ischemia, Alzheimer’s disease and Huntington’s disease (Faghihi et al., 2008; Johnson et al., 2010; Ng et al., 2013; Akula et al., 2014; Barry et al., 2014; Wang J. et al., 2017; Sarkar et al., 2018), and other diseases (Zhang et al., 2019). Interestingly, the expression of lncRNAs is found to be altered during cellular senescence, which represents a major risk factor for the development of many neurodegenerative diseases (Chakrabarti and Mohanakumar, 2016; Ghanam et al., 2017). A common theme emerging from these studies is that lncRNAs control the expression of nearby protein-coding genes *in cis* and that deregulation of this relationship could lead to brain diseases. The majority of these lncRNAs, which are considered to be expressed in the nervous system, have only been identified in genome-wide expression screens; intriguingly, their involvement in Parkinson’s disease (PD) is only beginning to be explored (Saracchi et al., 2014; Soreq et al., 2014; Ni et al., 2017; Marki et al., 2018).

In regards to PD, recent studies have identified lncRNAs that may alter the expression of PD-linked genes, such as *PINK1*, *UCH-L1*, *LRRK2*, and *SNCA.* In particular, NaPINK1, a human-specific lncRNA transcribed from the antisense orientation of the *PINK1* locus was found to stabilize PINK1 expression and its silencing resulted in decreased *PINK1* expression in neurons (Scheele et al., 2007; Chiba et al., 2009). Similarly, *UCHL1-AS1*, an antisense transcript of *UCH-L1*, was reported to enhance loading of *UCH-L1* mRNA to heavy polysomes for a more efficient translation, thus increasing protein synthesis (Carrieri et al., 2012). A subsequent study showed that *UCHL1-AS1* is a component of the *NURR1*-dependent dopaminergic gene network and is down-regulated in *in vitro* (MPP+) and *in vivo* (MPTP) toxin-induced models of PD (Carrieri et al., 2015). The activity of *UCHL1-AS1* is reported to be dependent on two functional domains: the overlapping region that defines target specificity and the inverted SINE element of B2 subclass (invSINEB2) that confers protein synthesis activation. These properties classified *UCHL1-AS1* as a representative member of a new functional class of natural and synthetic RNAs that increase protein synthesis named SINEUPs (Zucchelli et al., 2015). Based on the structure of this lncRNA, the authors designed synthetic SINEUPs targeting the endogenous PD-associated protein DJ-1, which proved to be active in different neuronal cell lines, suggesting that SINEUPs may represent valuable tools to increase synthesis of targeted proteins.

In addition, a recent report has shown that overexpression of the highly conserved neuron-specific lncRNA *MALAT1* (metastasis associated lung adenocarcinoma transcript 1), upregulated the expression of α-Synuclein (SNCA), whereas inhibition of MALAT1 downregulated SNCA expression only at the protein level rather than the mRNA level (Zhang et al., 2016). Finally, *HOTAIR* lncRNA (Hox transcript antisense intergenic RNA), transcribed from the HOXC locus, has been reported to be upregulated in the MPTP mouse model of PD. This upregulated HOTAIR was shown to increase the stability of *LRRK2* mRNA and to upregulate its expression, thus inducing dopaminergic neuronal apoptosis (Wang S. et al., 2017).

To investigate further the role of lncRNAs in PD pathogenesis, we have identified seven human lncRNA genes producing transcripts in close genomic proximity to PD-related genes, including *SNCA (PARK4)*, *LRRK2 (PARK8)*, *UCH-L1 (PARK5)*, *PINK1 (PARK6)*, *DJ-1 (PARK7)*, *GBA1*, and *MAPT*. These lncRNA genes have been formerly annotated by human genome consortia (ENCODE and FANTOM) and are thought to be potentially expressed in human cells. By detailed expression studies we showed that these lncRNAs are expressed in human dopaminergic SH-SY5Y cells and iPSC-derived human dopaminergic neurons, as well as in human post-mortem material derived from PD patients and controls. Importantly, our expression studies suggest that most of these lncRNAs are found to be under-expressed in the SN and three of them in the cerebellum of PD patients, compared to control subjects. Intriguingly, the majority of the aforementioned lncRNAs can be detected in human peripheral tissues, such as PBMCs and exosomes derived from CSF, suggesting their potential use as biomarkers. This line of research suggests a possible role of lncRNAs in PD pathogenesis or in neuronal responses associated with PD-related pathogenic effects.

## Materials and Methods

### Human Brain Specimens

**FIGURE 1:**
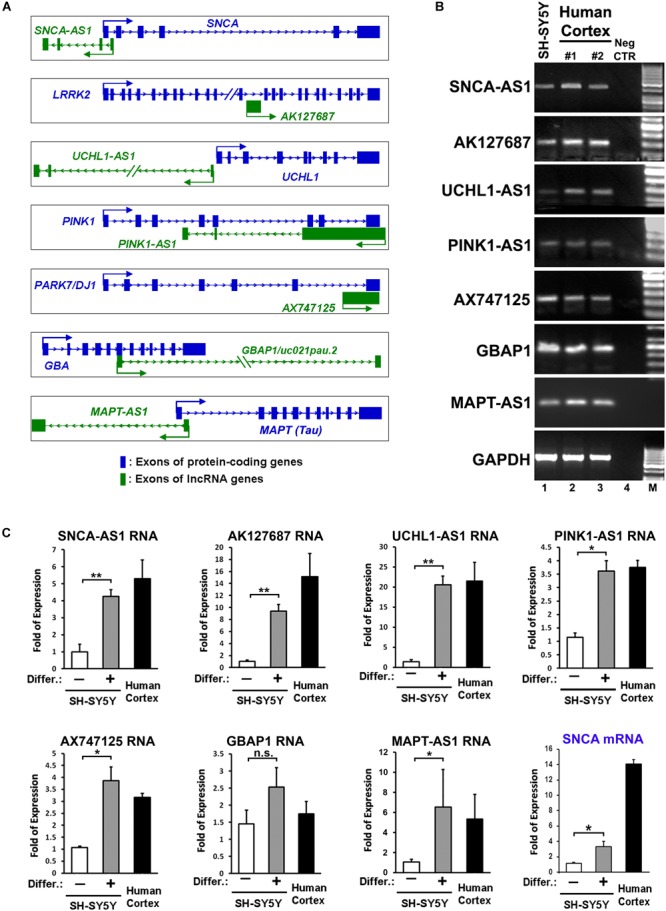
Identification and expression of lncRNAs within loci of protein coding genes related to Parkinson’s disease (PD). **(A)** Schematic representation of sense (*AK127687*, *AX747125*, *GBAP1*) and antisense (*SNCA-AS1*, *UCHL1-AS1*, *PINK1-AS1*, *MAPT-AS1*) lncRNA genes and their corresponding PD-linked protein coding genes. **(B)** Expression of the annotated lncRNA genes in human cortex and RA-differentiated or non-differentiated SH-SY5Y cells by conventional PCR analysis. As negative control a no-RT sample (Neg CTR) was used. As negative control **(C)** Relative RNA expression levels of the annotated lncRNA genes normalized to relative *GAPDH* levels, as well as relative SNCA mRNA levels, in SH-SY5Y cells treated with or without retinoic acid and in the human cortex from two independent individuals (*n* = 2), performed by qPCR analysis. All data are presented as the mean ± SEM from three independent experiments performed in triplicates (^∗^*p* < 0.05, ^∗∗^*p* < 0.01, two-tailed Student’s *t*-test).

A total of 17 cases (*n* = 8 non-demented controls; *n* = 9 PD cases) were included for the present study. Autopsy material encompassing the SN and the cerebellum was collected with full ethical permission, following donation by next of kin, and were kindly provided by the Parkinson’s Disease UK Brain Bank (PDUKBB). The referring specialist Neurologist in care of the patient locally made the clinical diagnosis, in accordance with internationally recognized criteria. Full clinical information and neuropathological reports are available upon request from the PDUKBB. The demographic details of these cohorts are shown in Table 1. Regarding the time from cardiac arrest to RNA isolation, this information is shown in Table 1. PMD (post-mortem delay) = 16.3 ± 6.7 for the Controls and 18 ± 6.97 for the PD subjects. The brain tissues arrived in dry ice and stored at -80°C, until further analysis. On the day of RNA isolation, the tissues were transferred on dry ice and Trizol reagent was added to all of them immediately, in order to avoid the thawing of the tissue. Study of these brains was performed following approval by the Ethical Committee of the Biomedical Research Foundation of the Academy of Athens. In preliminary experiments (presented in Figure 1) we also used autopsy material from the Netherlands Brain Bank, kindly provided to us by Dr Margarita Chrysanthou-Piterou from the Research Unit of Histochemistry and Electron Microscopy, University of Athens Medical School.

**Table 1:** Table presenting the demographic details for control (*n* = 8) and PD (*n* = 9) cohorts (n.a., non-applicable).

Sample	Sex	Age	Onset	Duration	PMD
		(years)	(years)	(years)	(hours)
Parkinson’s disease (*n* = 9)	5M:4F	81 ± 3.65	65.9 ± 7.99	15.1 ± 5.63	16.3 ± 6.7
Controls (*n* = 8)	4M:4F	82.1 ± 6.03	n.a.	n.a.	18 ± 6.97


### Isolation of Peripheral Blood Mononuclear Cells (PBMCs)

Patients with PD (*n* = 20) and age-matched healthy subjects (*n* = 20) were enrolled in the study from the 2nd Department of Neurology, Attikon University Hospital, Athens, Greece. Collection of samples was performed according to a protocol approved by the Scientific Council of “Attikon” Hospital, and all participating subjects signed informed consents. The diagnosis of idiopathic PD was made according to the criteria of Gelb et al. (1999). Motor function was evaluated with the Unified Parkinson Disease Rating Scale (UPDRS) III. Patients were also assessed for Hoehn & Yahr stage. Enrolled healthy controls were spouses and blood donors with no known family history of PD. The demographic details of these cohorts are shown in Table 2. PBMCs were isolated from whole blood by Biocoll Separating Solution (Biochrom AG) density gradient centrifugation. Briefly, blood samples were diluted with the same amount of Phosphate Buffer Saline (PBS, Gibco), layered on Biocoll and centrifuged (500 *g*, 20 min, 4°C). PBMCs were collected from the interface between plasma and Biocoll, washed twice with PBS, aliquoted and stored at -80°C until further analysis.

**Table 2:** Table presenting the demographic details for control (*n* = 20) and PD (*n* = 20) cohorts, in which the analysis of PBMCs was performed (n.a., non-applicable).

Sample	Sex	Age	Onset	Duration	UPDRS III
		(years)	(years)	(years)	(points)
Parkinson’s disease (*n* = 20)	13M:7F	64.95 ± 11.5	59.47 ± 11.4	5.47 ± 6.5	16.94 ± 9.2
Controls (*n* = 20)	9M:11F	63.43 ± 7.5	n.a.	n.a.	n.a.


### SH-SY5Y Cell Culture

SH-SY5Y cells were cultured in DMEM (Life Technologies) supplemented with 1% penicillin/streptomycin (Life Technologies) and 10% Fetal Bovine Serum (Life Technologies). Neuronal differentiation was performed with 10 μM all *trans* Retinoic acid (RA) as previously described (Vekrellis et al., 2009). The efficient differentiation of these cells into a neuronal-like phenotype was verified by immunostaining with the neuronal marker βIII-tubulin (Supplementary Figure 2). Proliferating and neuronally differentiated SH-SY5Y cells (7 days following initial RA addition) were used for this study.

### Human iPSC Culture and Neuronal Differentiation

**FIGURE 2:**
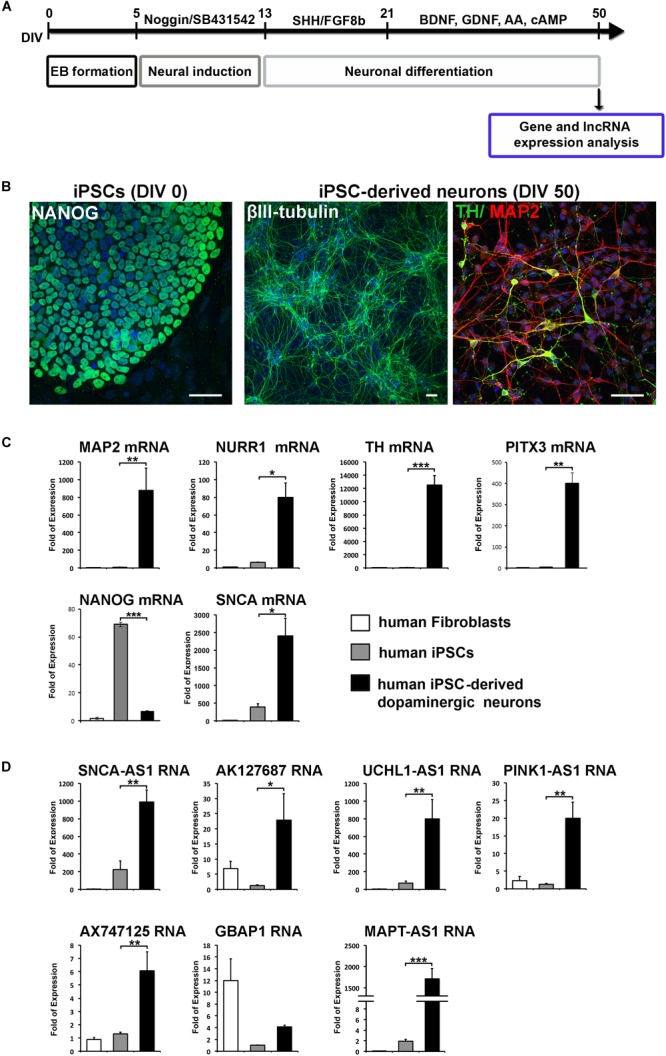
Specific expression of the identified lncRNAs in human iPSCs-derived dopaminergic neurons. **(A)** Schematic drawing of the protocol used for neuronal differentiation of iPSCs and timeline of analysis. At 50 DIV cells were either harvested for gene and lncRNA expression analysis or for phenotypic characterization by immunofluorescence staining. **(B)** Immunostaining of iPSCs for *NANOG* (pluripotency marker; green) and iPSC-derived neurons for *βIII-tubulin* (neuronal marker; green), *MAP2* (neuronal marker; red) and *TH* (dopaminergic neuronal marker; green). Cell nuclei are counterstained with DAPI (blue). Scale bar: 40 μm. **(C)** Real time RT-qPCR analysis showing the relative mRNA expression of *NANOG* (pluripotency), *SNCA*, *MAP2* (neuronal marker) and dopaminergic lineage markers *NURR1, TH*, and *PITX3*, normalized to relative *GAPDH* levels, in human fibroblasts, iPSCs and iPSC-derived neurons. **(D)** Real time RT-qPCR analysis showing the relative RNA expression of the identified lnc genes normalized to relative *GAPDH* levels in human fibroblasts, iPSCs and iPCSs-derived dopaminergic neurons. All data are presented as the mean ± SEM from three independent experiments performed in triplicates (^∗^*p* < 0.05, ^∗∗^*p* < 0.01, ^∗∗∗^*p* < 0.005, two-tailed Student’s *t*-test).

Skin fibroblasts derived from a control subject (age of skin biopsy 43 years old) were obtained through the MEFOPA study (MEndelianFOrms Of PArkinsonism). All study procedures were approved by the scientific council and ethical committee of Attikon University Hospital, Athens, Greece and the participant provided written informed consent. Fibroblasts were cultured in Dulbecco’s modified Eagle’s medium (DMEM, Life Technologies) supplemented with 10% fetal bovine serum (FBS, Life Technologies), 2 mM GlutaMax (Life Technologies) and 1% penicillin/streptomycin (Life Technologies). iPSCs were generated using the ‘Yamanaka’ reprogramming method by transducing human fibroblasts with retroviral vectors expressing the human cDNAs for OCT4, SOX2, KLF4 and C-MYC, as previously described (Takahashi et al., 2007). At least two iPSC lines were used for the current study. iPSCs were cultured on irradiated mouse embryonic fibroblasts (MEFs, Globalstem) in iPSC medium consisting of KnockOut DMEM (KO-DMEM, Life Technologies), 20% Knockout Serum Replacement (KSR, Life Technologies), 2 mM GlutaMax (Life Technologies), MEM Non-Essential Amino Acids (100x MEM NEAA, Life Technologies), 100 mM β-mercaptoethanol (Life Technologies) and 10 ng/ml human basic FGF (bFGF, Miltenyi). Medium was changed daily and cells were passaged as small clumps using collagenase (1 mg/ml, Life Technologies) every 3 to 6 days. iPSCs have been tested for their pluripotency *in vitro* and *in vivo*, as well as for their karyotype integrity (Kouroupi et al., 2017). Neural induction has been achieved by dual suppression of the SMAD signaling pathway using a combination of Noggin and SB431542 (Chambers et al., 2009). Dopaminergic differentiation was favored by culturing cells in B27/N2-medium containing human recombinant sonic hedgehog (SHH, R&D Systems) and murine recombinant fibroblast growth factor 8b (FGF-8b, R&D Systems) in the beginning, followed by brain-derived neurotrophic factor (BDNF, R&D Systems), glial cell-derived neurotrophic factor (GDNF, R&D Systems), ascorbic acid (AA, Sigma) and cyclic AMP (cAMP, Sigma) for terminal differentiation (Soldner et al., 2009). After almost 50 DIV of neuronal differentiation, cells were either harvested for RNA extraction or fixed for immunofluorescence staining (Figure 2A).

### Immunofluorescence Staining

Cells were fixed with 4% paraformaldehyde (Sigma-Aldrich) for 20 min at room temperature. Samples were blocked with 0.1% Triton X-100 (Sigma-Aldrich) and 5% donkey serum in PBS for 30 min and were subsequently incubated with primary antibodies against NANOG (marker for pluripotency; R&D Systems), βIII-tubulin (neuronal marker; Cell Signaling Technology), MAP2 (neuronal marker; Merck-Millipore) and tyrosine hydroxylase (TH, marker for dopaminergic lineage; Merck-Millipore) at 4°C overnight, followed by incubation with appropriate secondary antibodies (Molecular Probes, Thermo Fisher Scientific) conjugated to AlexaFluor 488 (green) or 546 (red), for at least 1 h at room temperature. Coverslips were mounted with ProLong Gold antifade reagent with DAPI (Cell Signaling Technology) and images were acquired using a Leica TCS-SP5II confocal microscope (LEICA Microsystems).

### RNA Extraction, cDNA Synthesis, and Real-Time PCR

Total RNA was extracted from the various cell types (SH-SY5Y, human fibroblasts, iPSCs, iPSC-derived neurons, PBMCs) and from human post-mortem material using the TRIzol^®^ Reagent (Ambion). Following digestion with DNase I, 1 μg of total RNA was used for first strand cDNA synthesis with the reverse transcription system (Invitrogen), according to manufacturer’s instructions. Primers were designed to anneal to sequences in exons on both sides of an intron. Only for AK127687 and AX747125, which are intron less, primers were inevitably designed within one exon. Quantitative PCR analyses were carried out in Light Cycler 96 (Roche) Real time PCR detection system by using FastStart Essential DNA Green Master (Roche). The nucleotide sequences of primer sets used are shown in Supplementary Table 1.

### CSF Sample Collection and Exosome Isolation

Lumbar puncture was performed at the L4-S1 interspace, between 9 and 12 AM after overnight fasting from two adult male non-PD subjects. Approximately 5 ml of CSF were drawn from these subjects and were collected in three polypropylene tubes. The first 2 ml-tube was used for routine diagnostic purposes. The following two tubes were immediately (within 30 min) centrifuged at 500 ×*g* for the removal of cells, aliquoted (0.5 ml) into 1 ml polypropylene tubes and stored at -80°C until analysis. They were thawed only once, just before the assay. The isolation of the exosomes was performed as previously described (Thery et al., 2006). Briefly, the collected CSF was centrifuged at 100,000 ×*g* for 2 h at 4°C. The supernatant (S100) was discarded and the pellet (P100) containing the externalized vesicles was reconstituted in 600 μl TRIzol^®^ Reagent and proceeded for RNA extraction as above.

### Statistical Analysis

All data are shown as mean ± standard error of mean (SEM). Statistical analysis was performed using GraphPad Prism 5.0. Non-parametric Mann–Whitney test or two-tailed Student’s *t*-test was used to assess the significance of differences between two groups, and non-parametric Spearman’s rho was used to assess the correlation between different variables of each sample. Probability values less than 0.05 (*p* < 0.05) were considered significant. The computed statistical power of the *t*-tests utilized to compare the data obtained in PD patients and Control subjects is 20.1% for the SN, 25.3% for the cerebellum and 26.3% for the analysis of PBMCs.

## Results

### Neuronal Expression of Human lncRNAs Associated With PD-Linked Genes

To investigate the involvement of lncRNAs in PD pathogenesis, we bioinformatically screened eight human genes, genetically linked to PD, for close proximity with lncRNA genes (based on publicly available data from UCSC genome browser, ENCODE and GTEX databases). In particular, we screened *SNCA*, *LRRK2*, *UCH-L1, PINK1*, *DJ-1*, *GBA1*, *MAPT*, and *PARK2 (PARKIN) genes*. Our analysis focused on genes linked to hereditary autosomal dominant PD, such as *SNCA (PARK4)*, *LRRK2 (PARK8)*, and *UCH-L1 (PARK5)*. According to genome wide association studies (GWASs) polymorphisms within the *SNCA* and *LRRK2* loci represent the most common genetic factors in the sporadic disease (Nalls et al., 2014; Hernandez et al., 2016). We have also chosen to screen *PINK1* (*PARK6*) (Valente et al., 2004; Marongiu et al., 2008), *PARKIN* (*PARK2*) (Kitada et al., 1998) and *DJ-1* (*PARK7*) (Bonifati et al., 2003; van der Merwe et al., 2015) because they are implicated in early-onset autosomal recessive PD. Finally, we have screened the *GBA1* gene, encoding for the lysosomal enzyme β-glucocerebrosidase (GCase) and the *MAPT* gene, encoding for the protein Tau, since both of them are considered susceptibility genes for the development of the disease (Davis et al., 2016). For *GBA1* in particular, genetic evidence has linked heterozygous *GBA1* mutations with PD, which in some populations, represent the strongest risk factor for PD, while an association of tagging SNPs within *SCARB2*, the gene encoding for the lysosomal membrane protein LIMP2 (responsible for the intra-lysosomal transport of GCase) with PD, has been also reported (Michelakakis et al., 2012).

Based on this analysis, we initially identified lncRNAs encompassing transcriptional units in close proximity (less than 0.5 kb genomic distance) to seven out of eight of these genes (with the exception of *PARK2*) designated as *SNCA-AS1*, *AK127687*, *UCHL1-AS1*, *PINK1-*AS1, *AX747125*, *GBAP1/uc021pau.2* and *MAPT-AS1*, respectively (Figure 1A). Of these, the *UCHL1-AS1*, the *PINK-AS1* and the *MAPT-AS1* have been previously reported to control the expression of *UCHL1* (Carrieri et al., 2012, 2015), *PINK1* (Scheele et al., 2007; Chiba et al., 2009), and *MAPT* (Coupland et al., 2016), respectively, but with the exception of *MAPT-AS1,* an analysis of their expression levels in correlation to the expression of the respective protein coding genes in human PD-affected areas was lacking. Interestingly, all these lncRNAs were expressed in human SH-SY5Y dopaminergic cells and in the human cortex (Figure 1B). The neuronal expression of three of these lncRNAs (*SNCA-AS1*, *UCHL1-AS1*, and *MAPT-AS1*; for the other four lncRNAs there were no available data) was also confirmed by analyzing the publicly available expression data from GTEX database (Supplementary Figure 1). To examine their pattern of expression during neuronal differentiation, we differentiated SH-SY5Y cells with the addition of 10 μM of RA, which resulted in increased expression of the mRNA levels of neuronal or dopaminergic markers *MAP2*, *NURR1* and *PITX3* (Supplementary Figure 2A) and of the protein levels of bIII-tubulin (Supplementary Figure 2B), as compared to proliferating cells (-RA). The expression levels of all these lncRNAs, except *GBAP1*, were significantly upregulated upon neuronal differentiation of dopaminergic SH-SY5Y cells (*p* < 0.05 for *PINK1-*AS1, *AX747125*, and *MAPT-AS1*; *p* < 0.01 for *SNCA-AS1*, *AK127687*, and *UCHL1-AS1*) to levels comparable to those detected in the human cortex, following the same trend as the *SNCA* mRNA levels, encoding for the neuronal protein SNCA (Figure 1C). The neuronal specificity of these lncRNAs was further evaluated in human iPSC lines and iPSC-derived neurons. In particular, skin fibroblasts of a healthy subject were reprogrammed to iPSCs, which have been extensively characterized for pluripotency and evaluated for their ability to terminally differentiate into dopaminergic neurons, as recently described (Kouroupi et al., 2017). Two iPSC lines were differentiated to dopaminergic neurons following a dual SMAD inhibition protocol (Chambers et al., 2009; Soldner et al., 2009) (Figure 2A). βIII-tubulin+ and MAP2+ neurons were efficiently generated from the iPSC lines, while almost 20% of MAP2+ neurons were found to express also tyrosine hydroxylase (TH; dopaminergic neurons) at 50 days *in vitro* (DIV; Figure 2B). To further verify the differentiation efficiency of iPSCs into dopaminergic neurons, we compared mRNA expression levels of neuronal (*MAP2*) and dopaminergic (*NURR1*, *TH, PITX3*) genes in fibroblasts, iPSCs and iPSC-derived neurons. All four transcripts were greatly induced in terminally differentiated cells (*p* < 0.05 for *NURR1*; *p* < 0.01 for *MAP2* and *PITX3*; *p* < 0.001 for *TH*), providing further evidence for the dopaminergic identity of the iPSC-derived neurons (Figure 2C). In contrast, mRNA levels of the pluripotency marker *NANOG* were significantly up-regulated during the reprogramming of fibroblasts to iPSCs and dramatically decreased in the iPSC-derived neurons (*p* < 0.001), further supporting the terminally differentiated state of the resulting neurons (Figure 2C). Furthermore, *SNCA* mRNA expression levels were found significantly augmented in the iPSC-derived dopaminergic neurons (*p* < 0.05) (Figure 2C). In agreement with the findings in the SH-SY5Y cells, all lncRNAs, except *GBAP1*, were highly increased in the iPSC-derived dopaminergic neurons (*p* < 0.05 for *AK127687*; *p* < 0.01 for *SNCA-AS1*, *UCHL1-AS1*, *PINK1-*AS1 and *AX747125*; *p* < 0.001 for *MAPT-AS1*) (Figure 2D). These data further suggest a correlation between the six (*SNCA-AS1*, *AK127687*, *UCHL-AS1*, *PINK1-AS1*, *AX747125*, and *MAPT-AS1*) out of the seven identified lncRNAs with neuronal differentiation and/or with the dopaminergic phenotype in particular.

### LncRNAs Associated With PD-Linked Genes Are Found Under-Expressed in PD Brains

**FIGURE 3:**
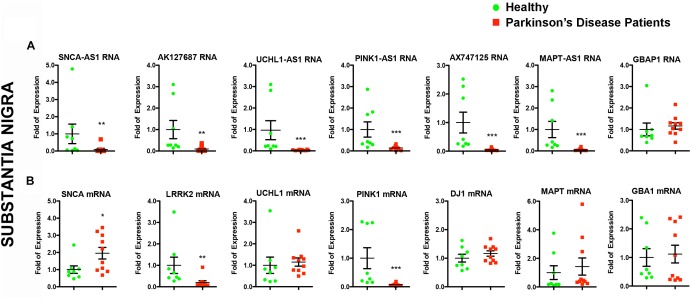
Relative RNA expression of lncRNAs and their corresponding protein coding genes in the SN of PD patients and controls. Real time RT-qPCR analysis of RNA expression of lncRNA genes **(A)** and their corresponding PD-linked genes **(B)** normalized relative to *GAPDH* levels, in the SN of PD patients and controls. All data are presented as the mean ± SEM (^∗^*p* < 0.05, ^∗∗^*p* < 0.01, ^∗∗∗^*p* < 0.001, Mann–Whitney *t*-test).

The elevated expression of six out of seven annotated lncRNAs in the iPSC-derived dopaminergic neurons prompted us to assess their relative expression levels in two human brain regions with different vulnerability in PD, the SN and the cerebellum derived from PD patients or age- and sex-matched control subjects (Table 1). Interestingly, all transcripts were expressed in the SN of healthy subjects, and all six lncRNAs exhibited remarkable under-expression in the SN of PD patients normalized relative to *GAPDH* levels (*p* < 0.01 for *SNCA-AS1*, *AK127687*; *p* < 0.001 for *UCHL1-AS1*, *PINK1-AS1*, *AX747125*, and *MAPT-AS1*) (Figure 3A). Only *GBAP1* transcript levels were not affected in the nigra of PD patients. To exclude that this dramatic decrease in the lncRNA expression merely represents the outcome of massive neuronal cell death in the SN of PD patients, we additionally normalized expression levels of these lncRNAs with the geometric mean of *NURR1-MAPT-GAPDH* levels, used as additional reference genes. Normalization of lncRNA expression levels versus the geometric mean of *NURR1-MAPT-GAPDH* levels gave similar results, since all six lncRNAs -except *GBAP1*- were still dramatically and significantly under-expressed in the SN of PD patients (*p* < 0.05 for *SNCA-AS1* and *UCHL1-AS1*; *p* < 0.01 for *AK127687*, *PINK1-AS1*, *AX747125*, and *MAPT-AS1*) (Supplementary Figure 3). To further exclude the possibility that this effect was non-specific, we have analyzed the expression of two PD-irrelevant lncRNAs, *hSNHG1*, and *hSNHG5*. As shown in Supplementary Figure 4, expression levels of both lncRNAs were similar between controls and PD nigral samples.

**FIGURE 4:**
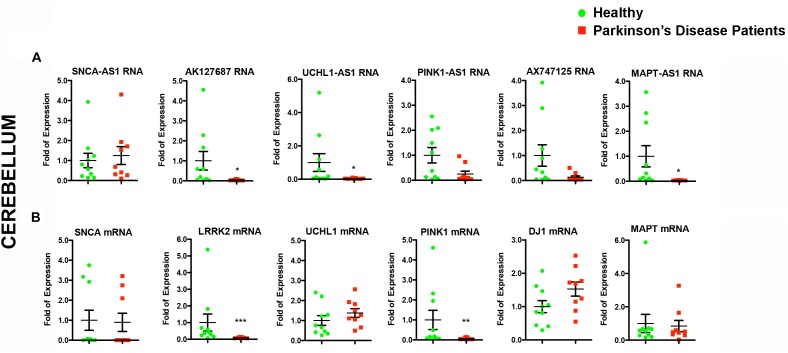
Relative RNA expression of lncRNAs and their corresponding protein coding genes in the cerebellum of PD patients and controls. Real time RT-qPCR analysis of RNA expression of lncRNA genes **(A)** and their corresponding PD-linked genes **(B)** in the cerebellum of the same PD and controls subjects. All data are presented as the mean ± SEM (^∗^*p* < 0.05, ^∗∗^*p* < 0.01, ^∗∗∗^*p* < 0.001, Mann–Whitney *t*-test).

Given that lncRNAs can functionally operate near their site of synthesis by regulating the transcription of their proximal gene(s) (Ponjavic et al., 2009; Wang and Chang, 2011; Qureshi and Mehler, 2012; Briggs et al., 2015), we analyzed the expression levels of the corresponding nearby genes. Interestingly, we found significant expression alterations for three of them in the SN of PD patients, namely for *SNCA* (*p* < 0.05), *LRRK2* (*p* < 0.01), and *PINK1* (*p* < 0.001) (Figure 3B). The mRNA levels of *LRRK2* and *PINK1* were found to be under-expressed in PD patients similar to their respective lncRNAs (Figure 3A,B). In contrast, *SNCA* mRNA levels were found increased in PD patients, exhibiting a discordant expression pattern with that of its antisense lncRNA (Figure 3A,B). To test whether such lncRNA transcript alterations may represent a hallmark of PD affected brain areas, we assessed the relative lncRNA expression in the cerebellum of the same PD patients and controls, an area that receives dopaminergic projection from the ventral tegmental area/SN and that has been shown to exhibit pathological changes following dopaminergic degeneration in PD patients and PD-like animal models (reviewed in Wu and Hallett, 2013). Three out of six lncRNAs [*AK127687* (*p* < 0.05), *UCHL1-AS1* (*p* < 0.05), and *MAPT-AS1* (*p* < 0.05)] were found decreased in the cerebellum of PD subjects (Figure 4A). Interestingly, the mRNA levels of *LRRK2* (*p* < 0.001) and *PINK1* (*p* < 0.01) followed a similar pattern to that in the nigra (Figure 4B). In contrast to the data obtained from the nigral tissues of the same PD patients, no changes were detected in the *SNCA-AS1* lncRNA or the *SNCA* mRNA levels in the cerebellar tissues derived from the same PD patients.

### RT-PCR Based Detection of LncRNAs in PBMCs and in CSF-Derived Exosomes

**FIGURE 5:**
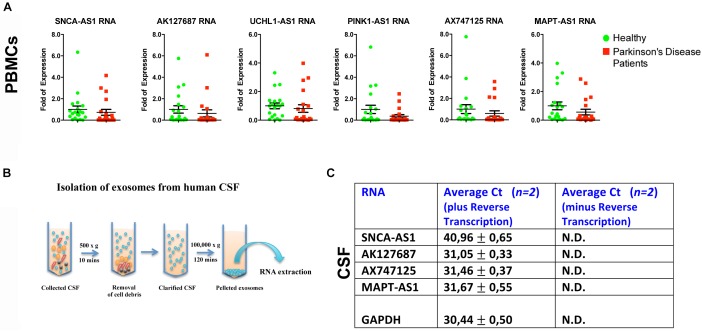
Relative expression of lncRNAs in PBMCs of PD patients and controls and in CSF-derived exosomes of non-PD subjects. **(A)** qPCR analysis of RNA expression of lnc genes normalized to relative *GAPDH* levels, in PBMCs derived from PD patients and controls. All data are presented as the mean ± SEM (Mann–Whitney *t*-test). **(B)** Cartoon depicting CSF exosome isolation and subsequent RNA extraction. **(C)** Detection (Average Ct values) of lncRNAs *SNCA-AS1*, *AK127687*, *AX747125*, and *MAPT-AS1* in CSF-derived exosomes of two non-PD subjects in comparison with the same samples without the reverse transcription step (negative control) or with the *GAPDH* (positive control) by Real time RT-qPCR analysis. N.D., not detected.

To further assess whether the aforementioned lncRNAs are also found dysregulated in peripheral tissues of PD patients, we analyzed their expression levels in PBMCs derived from sporadic PD patients and age- and sex-matched controls (Table 2). Real time RT-qPCR analysis showed that all six lncRNAs were expressed in human PBMCs (Figure 5A). However, the relative RNA expression of these lncRNAs, normalized to relative *GAPDH* expression levels, did not reveal statistically significant differences between PBMCs derived from PD patients and controls, although a slight trend for reduced expression in patients was observed for all lncRNAs tested (Figure 5A).

Importantly, by isolating exosomes from CSF derived from two non-PD subjects, we observed that that four out of the six lncRNAs *SNCA-AS1*, *MAPT-AS1*, *AK127687* and *AX747125*, could be detected in CSF-derived exosomes, as compared to the control samples (cDNAs prepared without reverse transcriptase) (Figure 5B,C). Three out of four were detected in levels similar to that of *GAPDH*, which has been previously reported to be found in exosomes (Xiao et al., 2012; Gezer et al., 2014; Yang et al., 2016; Barbagallo et al., 2018).

## Discussion

In recent years, the investigation into the role of lncRNAs in mammalian genome has uncovered an additional layer of gene regulation, suggesting their implication in many biological processes during brain development and progression of neurological diseases (reviewed by Ng et al., 2013; Barry, 2014; Briggs et al., 2015). The majority of lncRNAs previously associated with neurodegenerative diseases have only been identified in genome-wide expression screens and their functions remain largely unknown (Antoniou et al., 2014; Kraus et al., 2017; Pereira Fernandes et al., 2018). Understanding the role of lncRNAs in neurodegenerative diseases and specifically in PD could offer novel insights for elucidating the molecular mechanisms of the disease. To this end, in the current study we have found that, at least, six out of the seven identified lncRNAs exhibit elevated expression during directed differentiation of human iPSCs to dopaminergic neurons, or neuronal differentiation of SH-SY5Y cells, and that these lncRNAs are also expressed in the human cortex, SN and cerebellum in healthy individuals.

We should stress that our study focused mostly on genes linked to familial PD, such as *SNCA, LRRK2, UCH-L1*, *PINK1* and *DJ1*, as well as on the *GBA1* and *MAPT* genes, given that both of them are considered to be strong susceptibility genes. Beyond *SNCA* and *LRRK2*, whose contribution to PD pathogenesis is supported strongly by genetic studies, we have chosen to also study *UCH-L1* gene, although its genetic link to PD has been questioned (Lincoln et al., 1999; Maraganore et al., 1999, 2004; Healy et al., 2006). Our interest on *UCH-L1* was based on prior studies of our lab, showing that the S18Y polymorphic variant exerts antioxidant properties both *in vitro* (Kyratzi et al., 2008) and *in vivo* (Xilouri et al., 2012). Moreover, it should be noted that GWAS studies also suggested the contribution of other genes in the risk of developing PD, such as the human leukocyte antigen (*HLA*) locus, encoding MHCII molecules (Hamza et al., 2010; Alonso-Navarro et al., 2014; Jimenez-Jimenez et al., 2016), thus implicating innate immune function in disease pathogenesis. An analysis of a possible implication of altered levels of lncRNAs in close proximity to the *HLA* gene might be of interest for future studies.

Collectively, our data correlate the expression of the identified lncRNAs with human neuronal identity and indicate a possible role for these lncRNAs in the human brain. Considering that these lncRNAs are transcribed from genomic loci that have been previously genetically linked with PD pathogenesis, our observations raise the critical question of whether part of this genetic linkage or association is due to a potential involvement of these RNA molecules in PD pathogenesis. Accordingly, here we found that six of these lncRNAs are under-expressed in the SN and three out of six (*AK12768*7, *UCHL1-AS1,* and *MAPT-AS1*) in the cerebellum of PD patients.

Such data may support a potential correlation between the expression levels of these lncRNAs and PD pathogenesis. The SN is the area mainly involved in PD pathogenesis whereas the cerebellum is an area that may have certain roles in PD pathophysiology (Caligiore et al., 2016). In particular, neuroimaging studies in humans have found that PD patients have altered cerebellar activation during motor execution, motor learning and at rest (Wu and Hallett, 2013) In contrast to the SN, where SNCA-rich Lewy bodies are the main histopathological hallmark of the disease, no Lewy bodies are detected in the cerebellum of PD patients and some studies have reported decreased cerebellar *SNCA* mRNA levels (Fuchs et al., 2008). In our study, we have not detected alterations in the *SNCA* mRNA levels or the *SNCA-AS1* lncRNA levels in the cerebellum of PD patients, compared to controls. In contrast, in both SN and cerebellum, we detected decreased levels of *LRRK2* mRNA and of its corresponding lncRNA (*AK127687*), suggestive of a possible association between the expression levels of this lncRNA and PD pathogenesis, through influencing of LRRK2 levels. In agreement with our findings, decreased *LRRK2* mRNA levels in the cerebellum of PD patients have been also previously reported (Sharma et al., 2011). Moreover, in accordance with our hypothesis of a functional role of these dysregulated lncRNAs in controlling the expression of nearby protein coding genes, previous studies have reported the ability of *MAPT-AS1*, *PINK1-AS1*, and *UCHL1-AS1* to directly affect expression levels of *MAPT*, *PINK1*, and *UCHL1* genes, respectively (Scheele et al., 2007; Carrieri et al., 2012, 2015; Coupland et al., 2016). Interestingly, *MAPT-AS1* was also recently reported to be under-expressed in many brain regions of PD affected patients, including putamen, anterior cingulate cortex, visual cortex, and cerebellum (Coupland et al., 2016). These observations are in good agreement with our data and further confirm and validate our analyses.

We have carefully addressed the possibility that the under-expression of the six lncRNAs in the nigra of PD patients, observed in our study, might represent a non-specific result. As the expression of these lncRNAs showed a marked association with the establishment of a neuronal phenotype, our results could reflect nigral neuronal loss. However, the difference between PD patients and controls remained highly significant even following normalization with the geometric mean of *NURR1-MAPT-GAPDH*. Furthermore, mRNA levels of genes expressed selectively in neurons (*MAPT, UCH-L1*) showed no difference between the nigral tissues of PD and control brains. Moreover, we have not observed a generalized decrease of such lncRNA’s expression in degenerating SH-SY5Y cells exposed to the neurotoxin MPP^+^ (data not shown), suggesting again that the findings in PD brains do not reflect non-specific neurodegeneration. Therefore, we believe that our findings truly mirror under-expression of the studied lncRNAs in remaining nigral neurons or in the largely intact cerebellar neurons in the context of PD.

As lncRNAs can act at both directions of transcriptional regulation, either activating or repressing gene expression (Wang and Chang, 2011; Guttman and Rinn, 2012; Flynn and Chang, 2014), such lncRNAs might represent key components for the regulation of PD-linked genes. This may be especially important regarding the regulation of *SNCA* expression, which is strongly linked to both idiopathic and genetic forms of PD. However, based on our experimental evidence, we cannot exclude the possibility that these differences could represent secondary effects of other pathogenetic mechanisms underlying PD progression, i.e., chromatin reorganization of the affected loci. To our knowledge, this is the first time that such a correlation between PD-affected brain regions and specific lncRNAs is established, although further experimentation is needed to understand the precise molecular mechanism of their action.

It is important to note that these lncRNAs were detected in peripheral tissues such as PBMCs and CSF (Figure 5), even though no statistically significant differences were observed between the PBMCs of controls and PD patients. Such data do not rule out the possibility that a difference might exist, but that a larger number of samples is required so that the trends observed in the present analyses reach statistical significance. We have only performed proof of principle analysis in human CSF exosomes, establishing that four out of the six lncRNAs are detected in this biological material. Clearly, further studies will be needed to investigate whether the expression levels of these lncRNAs in CSF exosomes, reflecting presumably their total brain levels, are different between PD and controls. Moreover, another approach would be to isolate neuronally-derived exosomes from plasma of PD subjects and controls and compare the expression levels of lncRNAs, as it was previously reported for levels of *SNCA* (Shi et al., 2014). It is expected that this neuronal enrichment may lead to more marked differences in PD vs. controls, probably reflecting specific brain-related changes. A growing body of evidence suggests that exosomal lncRNAs circulating in serum or urine may represent potential diagnosis biomarkers and prognosis indicators in cancer (Dragomir et al., 2018; Gramantieri et al., 2018; Sun et al., 2018); whether such a role could be attributed to neuronally-derived exosomes remains to be explored. The emerging importance of lncRNAs as diagnostic tools is further supported by recent studies that utilized RNA-sequencing data analysis to identify differential expression levels of lncRNAs in leukocytes of PD patients compared to controls, as well as in the human SN of PD patients (Soreq et al., 2014; Ni et al., 2017).

Collectively, our data suggest that lncRNAs are associated with PD pathogenesis and may thus represent novel targets for the diagnosis and/or treatment of PD and related disorders.

## Author Contributions

ME, MX, and PP performed experiments, analyzed the data and prepared the manuscript. GK and RM generated and characterized human iPSCs and iPSC-derived neurons, AV, NP, and VK performed experiments. LS, MX, and PP designed and supervised the project. PP conceived the original idea of this manuscript and initially identified the selected lncRNAs. All authors critically read and approved the manuscript.

## Conflict of Interest Statement

The authors declare that the research was conducted in the absence of any commercial or financial relationships that could be construed as a potential conflict of interest.

## Supplementary Material

FIGURE S1 Expression analysis of lncRNAs *SNCA-AS1*, *UCHL1-AS1*, and *MAPT-AS1* in various human tissues and organs, based on GTEX database. The expression data for the three lncRNAs were analyzed and downloaded from the publicly available GTEX webpage, https://gtexportal.org/home/.

FIGURE S2 Confirmation of neuronal differentiation of SH-SY5Y cells with the addition of RA. **(A)** Relative mRNA expression levels of *MAP2*, *NURR1*, and *PITX3* in the absence (-) or presence (+) of RA, performed by RT-qPCR analysis. All data are presented as the mean ± SEM from three independent experiments performed in triplicates (^∗^*p* < 0.05, ^∗∗^*p* < 0.01, ^∗∗∗^*p* < 0.001, two-tailed Student’s *t*-test). **(B)** Immunofluorescence analysis of the same cells under the same conditions with the anti-bIII-tubulin antibody (TuJ1), as indicated.

FIGURE S3 Relative RNA expression of PD-linked lncRNAs in the *Substantia Nigra* of PD patients and controls normalized to the geometric mean of *GAPDH-NURR1-MAPT l*evels. Real time RT-qPCR analysis of RNA expression of lncRNA genes normalized to the geometric mean of *GAPDH-PPI1A-RPLI3A* levels in the *Substantia Nigra* of controls and PD patients. All data are presented as the mean ± SEM from three independent experiments performed in triplicates.

FIGURE S4 Relative RNA expression of non PD-linked lncRNAs in the *Substantia Nigra* of PD patients and controls. Real time RT-qPCR analysis of hSNHG1 and hSNHG5 lncRNA RNA expression normalized to GAPDH in the *Substantia Nigra* of controls and PD patients. All data are presented as the mean ± SEM from three independent experiments performed in triplicates.

## Abbreviations

CSF: cerebrospinal fluid
iPSCs: induced pluripotent stem cells
lncRNAs: long non-coding RNAs
PBMCs: peripheral blood mononuclear cells
SN: *Substantia Nigra*
