# Supplementary figures and images for: Long Non-coding RNAs Associated With Neurodegeneration-Linked Genes Are Reduced in Parkinson’s Disease Patients
Source: Front Cell Neurosci. 2019 Feb 22;13:58. doi: 10.3389/fncel.2019.00058 (PMC6396023; doi:10.3389/fncel.2019.00058)

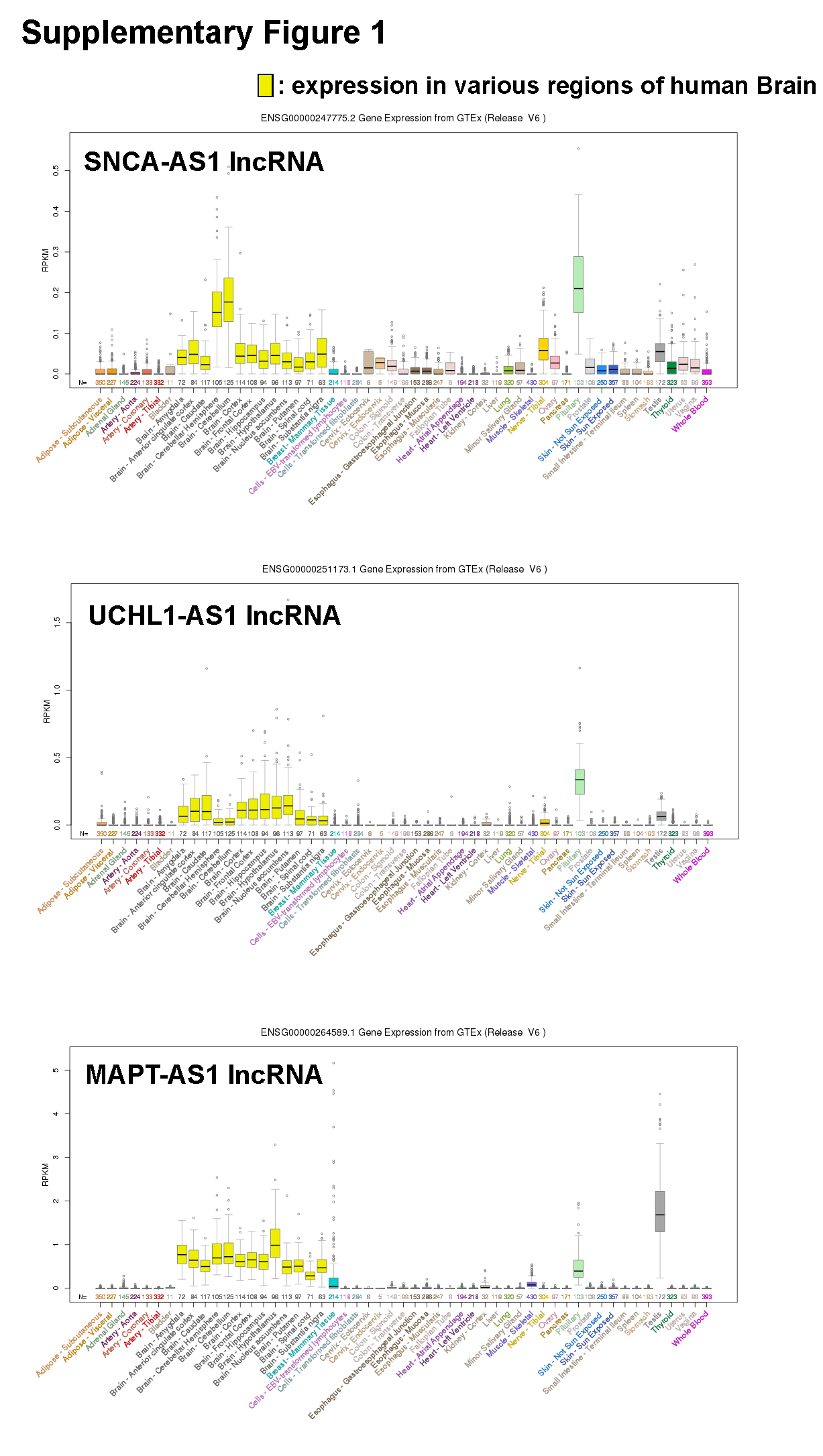

Supplement: FIGURE S1 — Expression analysis of lncRNAs SNCA-AS1, UCHL1-AS1, and MAPT-AS1 in various human tissues and organs, based on GTEX database. The expression data for the three lncRNAs were analyzed and downloaded from the publicly available GTEX webpage, https://gtexportal.org/home/. [file Image_1.TIF]

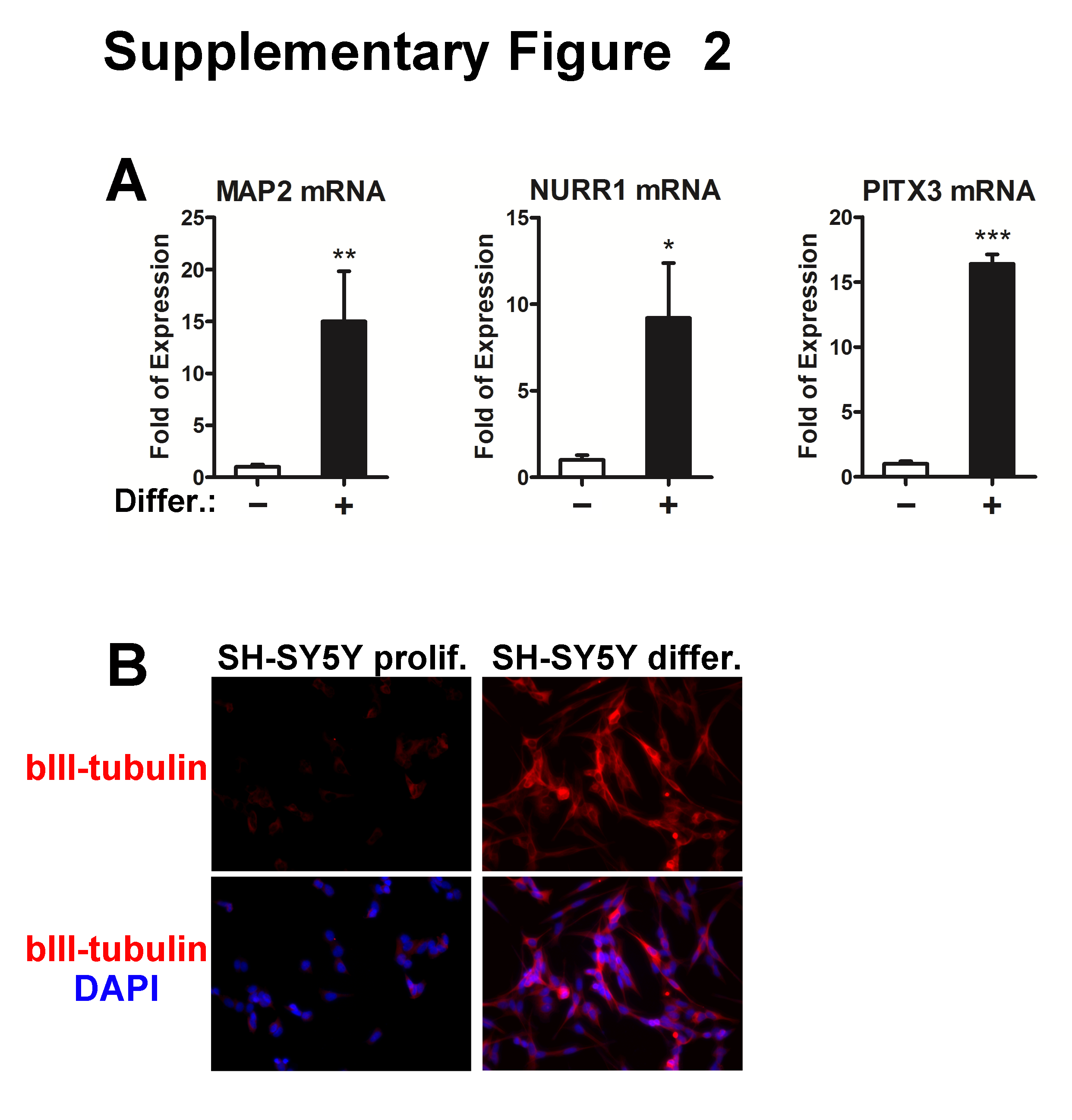

Supplement: FIGURE S2 — Confirmation of neuronal differentiation of SH-SY5Y cells with the addition of RA. (A) Relative mRNA expression levels of MAP2, NURR1, and PITX3 in the absence (-) or presence (+) of RA, performed by RT-qPCR analysis. All data are presented as the mean ± SEM from three independent experiments performed in triplicates (∗p < 0.05, ∗∗p < 0.01, ∗∗∗p < 0.001, two-tailed Student’s t-test). (B) Immunofluorescence analysis of the same cells under the same conditions with the anti-bIII-tubulin antibody (TuJ1), as indicated. [file Image_2.TIF]

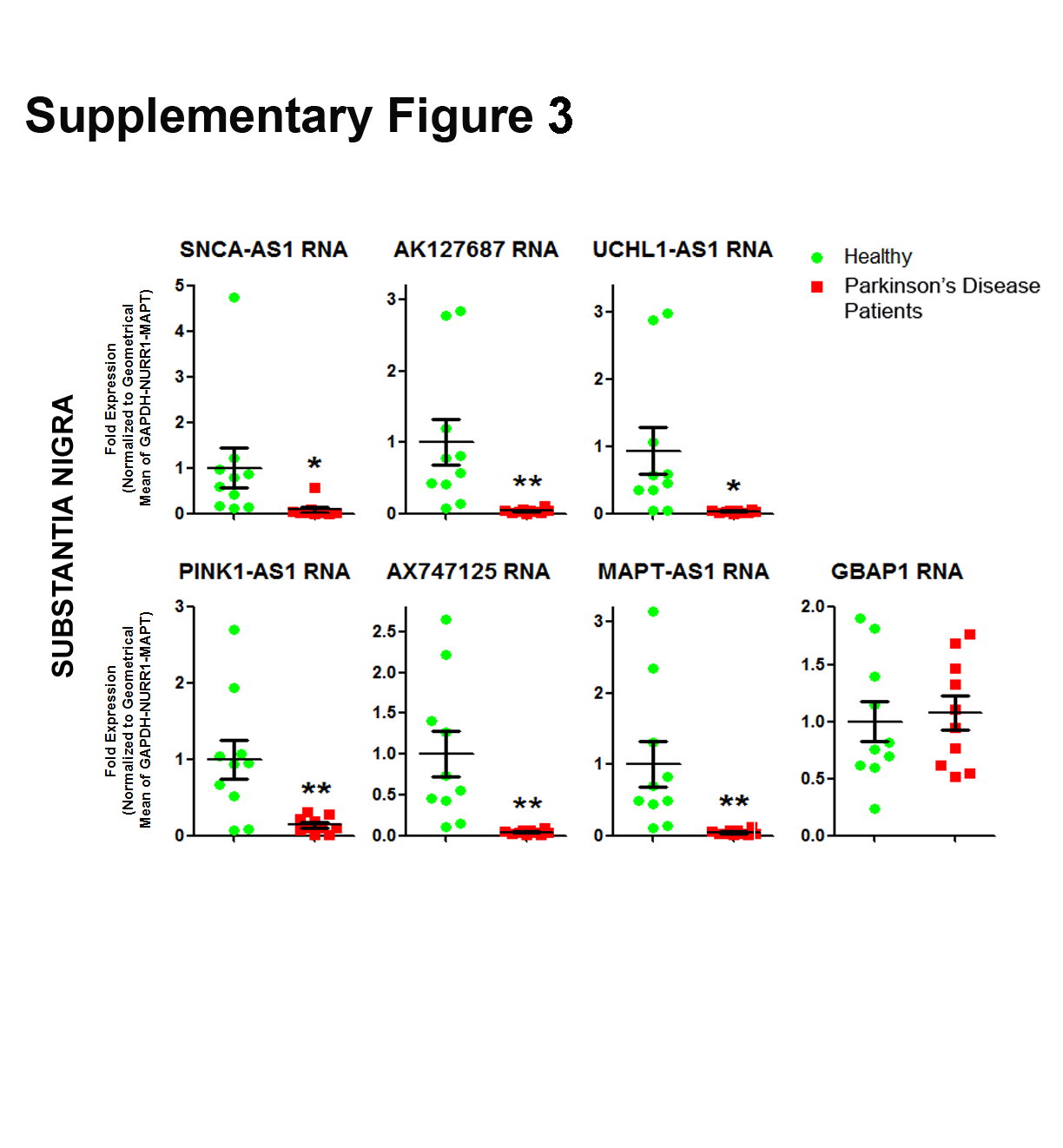

Supplement: FIGURE S3 — Relative RNA expression of PD-linked lncRNAs in the Substantia Nigra of PD patients and controls normalized to the geometric mean of GAPDH-NURR1-MAPT levels. Real time RT-qPCR analysis of RNA expression of lncRNA genes normalized to the geometric mean of GAPDH-PPI1A-RPLI3A levels in the Substantia Nigra of controls and PD patients. All data are presented as the mean ± SEM from three independent experiments performed in triplicates. [file Image_3.TIF]

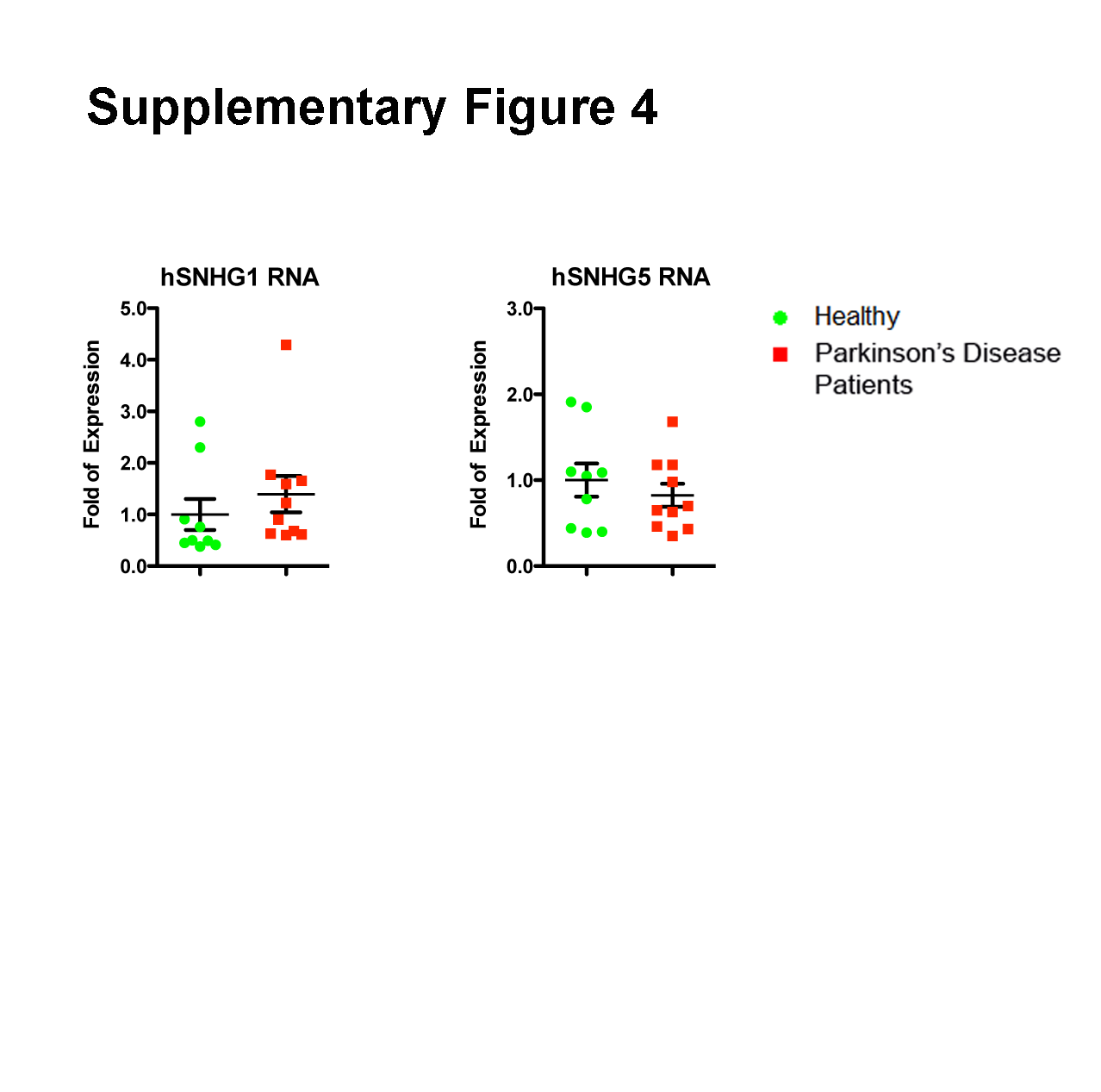

Supplement: FIGURE S4 — Relative RNA expression of non PD-linked lncRNAs in the Substantia Nigra of PD patients and controls. Real time RT-qPCR analysis of hSNHG1 and hSNHG5 lncRNA RNA expression normalized to GAPDH in the Substantia Nigra of controls and PD patients. All data are presented as the mean ± SEM from three independent experiments performed in triplicates. [file Image_4.TIF]
