# Supplementary material for: Long Non-coding RNAs Associated With Neurodegeneration-Linked Genes Are Reduced in Parkinson’s Disease Patients
Source: Front Cell Neurosci. 2019 Feb 22;13:58. doi: 10.3389/fncel.2019.00058 (PMC6396023; doi:10.3389/fncel.2019.00058)
Supplement: Supplementary file 5 [file Table_1.DOC]

| **Gene** | **Forward Primer** | **Reverse Primer** |
| --- | --- | --- |
| GAPDH | CCTCTGACTTCAACAGCGACAC | AGCCAAATTCGTTGTCATACCAG |
| SNCA | GCCCAAGAAATACACGCAAC | ATTGATGGGAAGGCATCAGA |
| PINK1 | CCCAAGCAACTAGCCCCTC | GGCAGCACATCAGGGTAGTC |
| LRRK2 | AATGAGCTTCCTCACGCAGT | TACAAAGCCACTTGGGTTCC |
| UCHL1 | AATGTCGGGTAGATGACAAGGT | GGCATTCGTCCAATCAAGTTCATA |
| MAPT | CCAAGTGTGGCTCATTAGGCA | CCAATCTTCGACTGGACTCTGT |
| DJ1 (PARK7) | AGCTCTGGTCATCCTGGCTA | CCTGCAACGGTGACCTTAAT |
| NURR1 | TCGACATTTCTGCCTTCTCCTG | GGTTCCTTGAGCCCGTGTCT |
| TH | TGTCTGAGGAGCCTGAGATTCG | GCTTGTCCTTGGCGTCACTG |
| MAP2 | GAGAATGGGATCAACGGAGA | CTGCTACAGCCTCAGCAGTG |
| GBA | TGCATCCTGCCTTCAGAGTC | GATAGAGGATCCACGTCGGC |
| NANOG | AGTCCCAAAGGCAAACAACCCACTTC | TGCTGGAGGCTGAGGTATTTCTGTCTC |
| SNCA-AS1 | GCCCAAGAAATACACGCAAC | ATTGATGGGAAGGCATCAGA |
| AX747125 | TTGCCAAGAAATAGCCAACC | GGGTATGCATGTGAGTGTGC |
| AK127687 | TGCTTGCAATTCCTTGACAG | TCATCACGGCTAGAATGCAG |
| UCHL1-AS1 | GTCGTCTGCCCAAAACTAGC | AAGGTGGACACCAGCTCATC |
| MAPT-AS1 | ATGCTGGATTCTGAGCCACT | GGCTTCAAATCCACCTCTCA |
| PINK1-AS1 | TGGAGAGGAAGCCACGATAC | TCTTTCCTCGCATCTCCTGT |
| GBAP1 | ACTGGGCAAAGGTGGTACTG | GGTTTGTGATGATGCTGTGG |
| hSNHG1 | TAACCTGCTTGGCTCAAAGG | CAGCCTGGAGTGAACACAGA |
| hSNHG5 | CACAGTGGAGCAGCTCTGAA | TCACTGGCTACTCGTCCACA |
| PITX3 | GAGCTAGAGGCGACCTTCC | CCGGTTCTTGAACCACACCC |

**Supplementary Table 1.** Table presenting nucleotide sequences of primer sets used in this study.
